# Decreased modular segregation of the frontal–parietal network in major depressive disorder

**DOI:** 10.3389/fpsyt.2022.929812

**Published:** 2022-07-22

**Authors:** Zhihui Lan, Wei Zhang, Donglin Wang, Zhonglin Tan, Yan Wang, Chenyuan Pan, Yang Xiao, Changxiao Kuai, Shao-Wei Xue

**Affiliations:** ^1^Center for Cognition and Brain Disorders, The Affiliated Hospital of Hangzhou Normal University, Hangzhou, China; ^2^Institute of Psychological Science, Hangzhou Normal University, Hangzhou, China; ^3^Zhejiang Key Laboratory for Research in Assessment of Cognitive Impairments, Hangzhou, China; ^4^Jing Hengyi School of Education, Hangzhou Normal University, Hangzhou, China; ^5^Affiliated Mental Health Center and Hangzhou Seventh People’s Hospital, Zhejiang University School of Medicine, Hangzhou, China

**Keywords:** frontal–parietal network, major depressive disorder, modular segregation, participation coefficient, fMRI

## Abstract

Major depressive disorder (MDD) is a common psychiatric condition associated with aberrant large-scale distributed brain networks. However, it is unclear how the network dysfunction in MDD patients is characterized by imbalance or derangement of network modular segregation. Fifty-one MDD patients and forty-three matched healthy controls (HC) were recruited in the present study. We analyzed intrinsic brain activity derived from resting-state functional magnetic resonance imaging (R-fMRI) and then examined brain network segregation by computing the participation coefficient (PC). Further intra- and inter-modular connections analysis were preformed to explain atypical PC. Besides, we explored the potential relationship between the above graph theory measures and symptom severity in MDD. Lower modular segregation of the frontal–parietal network (FPN) was found in MDD compared with the HC group. The MDD group exhibited increased inter-module connections between the FPN and cingulo-opercular network (CON), between the FPN and cerebellum (Cere), between the CON and Cere. At the nodal level, the PC of the anterior prefrontal cortex, anterior cingulate cortex, inferior parietal lobule (IPL), and intraparietal sulcus showed larger in MDD. Additionally, the inter-module connections between the FPN and CON and the PC values of the IPL were negatively correlated with depression symptom in the MDD group. These findings might give evidence about abnormal FPN in MDD from the perspective of modular segregation in brain networks.

## Introduction

Major depressive disorder (MDD) is a protect common psychiatric illness characterized by persistent low mood, loss of interest, vegetative symptoms and impaired cognitive function (1). MDD is highly prevalent affecting millions of people worldwide (2). As a leading cause of disability for recent several decades, MDD is a major contributor to the overall global disease burden (3). However, the precise pathophysiological mechanism underlying MDD is still unclear. Functional magnetic resonance imaging (fMRI), as a non-invasive technique, has provided a new insight for exploring brain mechanism using depicting universal patterns of brain activity and brain networks (4–6). With the help of fMRI, the human brain has been found as an optimized network framework which consists of highly specialized and relatively independent modules (7–9). In this framework, the connections between modules are sparse and the connections within the modules are tight (10), bringing about a modular segregation pattern which maintains the balance between functional specialization and integration, and then underlies individual cognitive processing and behavior performance (11–13). Convergent evidences using fMRI have demonstrated that this balance has been broken in MDD including lower modular segregation and network dysfunction (14–16). Specifically, changed intra-modular connections of the default mode network (DMN) regulating internally orientation is linked to pathological introspection symptom in MDD (17), the fronto-parietal network (FPN) and cingulopercular network (CON) involved in top-down process are associated with emotional dysregulation and poor concentration (18), and the salience network (SN) monitoring salient stimuli plays a crucial role in emotional control (19, 20). Likewise, imbalanced inter-modular connections between these networks are implicated in the expression of various characteristics underlying MDD. For example, a prior resting-state fMRI study has documented aberrant connectivity of the FPN with the SN and DMN leads to imbalance of externally attention process and internally self-reference and furthermore gives rise to emotional dysregulation (21). Another study based on meta-analysis reported hyperconnectivity between the SN and DMN is associated with overreaction to negative stimuli, which results in distorted information processing (22). Reduced connectivity between the executive network (EN) and DMN contributes to higher cognitive dysfunction (23). Although these studies regarded MDD as a “network disease,” modular segregation of the whole-brain functional networks in participants with MDD is not yet fully clear.

Graph theory analysis is an effective method to describe modular segregation of brain networks (24–26). In this method, the whole brain regions and the relationships between these regions are mapped as nodes and edges, respectively, and the graphics composed of these distributed nodes and edges are used to describe network topology structure (27). For the sake of evaluating modular segregation in brain networks, a powerful graph theory metric participant coefficient (PC) was utilized. The PC enables quantification of inter- and intra-module connections of brain networks (28). Applying the PC, a variety of previous neuroimaging studies have demonstrated aberrant modular segregation in various neuropsychiatrical or neuropsychological disorders. For example, Wang et al. reported that children showed higher intra-modular connections and lower inter-module connections with age (24). Zhou et al. (29) found that participants with internet game disorder had reduced intra-modular connections within the DMN and FPN relative to healthy controls (HC). Furthermore, a resting-state fMRI study also documented disrupted modular organization in traumatic brain injury (30). Therefore, graph theory analysis may contribute to uncovering abnormal modular segregation of brain network in MDD patients.

In the present study, we aimed to explore the modular segregation of brain networks in MDD using graph theory analysis. We firstly computed the mean PC in each module to identify which module or modules drove the pathological symptom of MDD. We then analyzed the number of intra- and inter-modular connections. Moreover, the PC of each node in module exhibiting abnormal modular segregation was obtained. Finally, Pearson correlation analysis were employed to investigate potential relationship between the above graph theory measures and symptom severity in the MDD group. Based on prior studies (22, 31), we hypothesized that (1) modular segregation of the whole-brain networks would be disrupted between the MDD and control groups; (2) graph theory properties with between-group differences would correlate with MDD symptom scores.

## Materials and methods

### Participants

Ninety-four participants were collected in the present study, including 51 MDD patients (age: 25.80 ± 8.80 years; gender: 15 males/36 females) and 53 gender-, age-, and handedness-matched HC (age: 29.42 ± 12.56 years; gender: 16 males/27 females). These participants were recruited from the Department of Psychiatry at the Affiliated Hospital of Hangzhou Normal University and the Department of Psychiatry of Hangzhou Seventh People’s Hospital.

MDD patients were diagnosed based on the Diagnostic and Statistical Manual of Mental Disorders, Fourth Edition (DSM-IV) criteria using the Mini-Neuropsychiatric International Interview (MINI). Symptom severity of MDD was evaluated according to 24-item Hamilton Depression Rating Scale (HAMD). All MDD patients did not take any antidepressant treatment or drug treatment for 2 months before taking part in the experiment. All procedures were approved by the local Institutional Review Board of Hangzhou Normal University and were conducted in accordance with the Declaration of Helsinki. Written informed consent was obtained from all participants or their guardian before participation.

### Imaging acquisition and preprocessing

**FIGURE 1:**
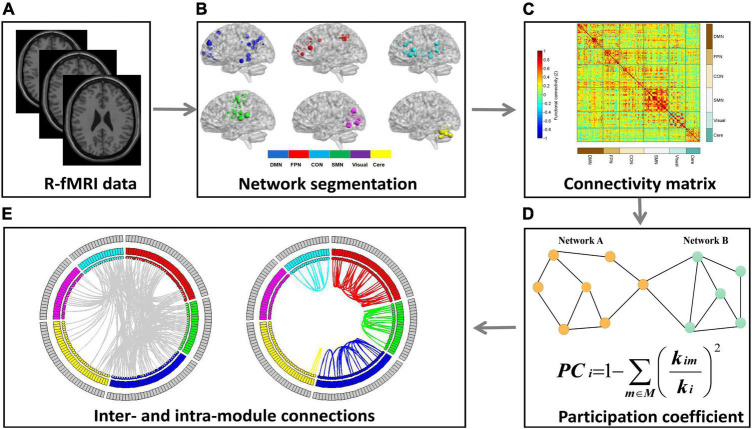
The schematic illustration of processing steps. This figure showed the data analysis pipeline of the present study. **(A)** Resting-state fMRI (R-fMRI) images were acquired from all subjects. **(B)** The Dosenbach 160 regions were used as network nodes of the whole brain and the 160 nodes were parcellated into six functional modules. **(C)** We conducted Pearson’s correlation analysis between each pair of nodes to construct a 160 × 160 correlation matrix for each participant. **(D)** The mean participation coefficient (PC) for each module was calculated to quantify the modular segregation. **(E)** The number of intra- and inter-module connections were calculated to explore the reasons for PC abnormalities among the two groups.

Imaging scanning was performed using a 3.0 T Discovery MR 750 scanner (General Electric, Waukesha, WI, United States) at the Center for Cognition and Brain Disorders of Hangzhou Normal University. All participants were asked to relax and keep their eyes closed without falling asleep before the scanning. Functional images (Figure 1A) were acquired using a T2*-weighted gradient-echo EPI pulse sequence with repetition time (TR) = 2,000 ms, echo time (TE) = 22 ms, flip angle (FA) = 77°, field of view (FOV) = 240 × 240 mm^2^, matrix = 96 × 96, 2.5 mm isotropic spatial resolution with 42 slices and 240 volumes. High-resolution T1-weighted structural images in the sagittal orientation were collected with fast spoiled gradient echo (3D FSPGR) sequence using the following parameters: TR = 9 ms, TE = 3.66 ms, FA = 13°, FOV = 240 × 240 mm^2^, matrix = 300 × 300, 0.8 mm isotropic voxels, 176 slices without interslice gap.

The Data Processing Assistant for Resting-State fMRI (DPARSF)^1^ software (32) was used to preform preprocessing and their detailed steps included: (1) discarding the first ten functional volumes for the scan environmental adaptation; (2) slice time correction on the remaining images; (3) implementing head motion correction (translation < 2 mm or rotation < 2°); (4) nuisance covariate regression (six motion parameters, white matter signal and cerebrospinal fluid signal); (5) normalization into the Montreal Neurological Institute (MNI) template using the transformation derived from T1 segmentation, and resampling at 3 × 3 × 3 mm^3^; (6) smoothing with a 6 mm full-width half-maximum isotropic Gaussian kernel; (7) detrending; (8) temporal filtering (bandpass, 0.01–0.1 Hz).

### Brain network construction and graph theory analysis

We adopted a functional brain template for data analysis according to a prior study (33). This template divided the brain into 160 regions which covered cerebral and cerebellar regions. We extracted the average time series from the 160 regions of interest (ROI) with 3 mm diameter and a 160 × 160 correlation matrix for each participant was obtained by performing Pearson’s correlation analysis on each ROI pair. Following prior studies (34, 35), a scarcity threshold of 15% was adopted to ensure that the number of network edges was the same across participant. Graph theory analysis was implemented by the Gretna software. Based on a predefined parcellation template (33), the 160 ROIs were grouped into six functional modules, which were labeled as the default-mode network (DMN), FPN, cingulo-opercular network (CON), sensorimotor network (SMN), visual network (Visual), and the cerebellum (Cere). The PC is an effective approach to evaluate modular segregation (28). For a given node *i* in the module *m*, PC was calculated as $P\,C\,i=1-\sum_{m\in M}{\left(\frac{k\,i\,m}{k\,i}\right)}^{2}$, where *M* is the set of modules, *k*_*im*_ is the number of connections between node *i* and other nodes in the module *m*, and *k*_*i*_ is the total number of connections of node *i*. The PC quantifies the patterns of inter- and intra-module connectivity of node *i*. Specifically, for a node *i* in the module *m*, higher *PC*_*i*_ value indicates lower connections within the module *m* and higher inter-module connections (lower modular segregation) while lower *PC*_*i*_ value suggests higher connections within the module *m* and lower inter-module connections (greater modular segregation). For each participant, the mean PC in each module was calculated by averaging the PC values of all nodes belonging to the module, so as to uncover which module causes the core symptom of MDD. In addition to PC, we calculated the number of intra-module connections in each module and the number of inter-module connections in each pair of modules. The number of inter-module connections was obtained by calculating the sum of connections between all nodes in a certain module and all nodes in another module. Finally, in order to explore abnormal modular segregation at node level, we calculated the PC of each node in the module showing atypical modular segregation.

### Statistical analysis

We performed two-sample *t*-test to compare between-group differences in the mean PC of each module, the number of intra-module connections and inter-module connections, and the PC of each node in the module with aberrant modular segregation. The normal test analysis showed that each item of data analyzed conformed to a normal distribution. Bonferroni correction was conducted for multiple comparisons correction and the significant threshold was set at α = 0.05/6 (six measures) = 0.0083. The chi-square test assessed the gender difference between the MDD and HC group. In order to measure the relationship between graph theory metrics and clinical data, we carried out Pearson correlation analysis in the MDD group.

## Results

Demographic and clinical data are listed in Table 1. There were no significant differences in gender (*p* = 0.43) and age (*p* = 0.11) between the MDD and HC groups. The MDD group exhibited significantly higher HAMD scores (*p* < 0.001) compared with the HC group.

**TABLE 1:** Demographic and clinical data.

	MDD (Mean ± *SD*)	HC (Mean ± *SD*)	*t*/χ^2^-value	*P*-value
Gender (male/female)	51 (15/36)	43 (16/27)	0.64	0.43^b^
Age (years)	25.80 ± 8.80	29.42 ± 12.56	−1.64	0.11^a^
Handedness (R/L)	51/0	43/0		
Mean FD	0.05 ± 0.02	0.06 ± 0.03	−1.58	0.12^a^
HAMD	28.55 ± 6.84	1.35 ± 1.38	27.73	<0.001^a^
Duration of illness (months)	8.08 ± 14.09			

MDD, major depressive disorder; HC, healthy controls; SD, standard deviation; R, right; L, left; FD, framewise displacement; HAMD, 24-item Hamilton Depression Rating Scale.^a^Two-sample t-test.^b^Chi-square test.

**FIGURE 2:**
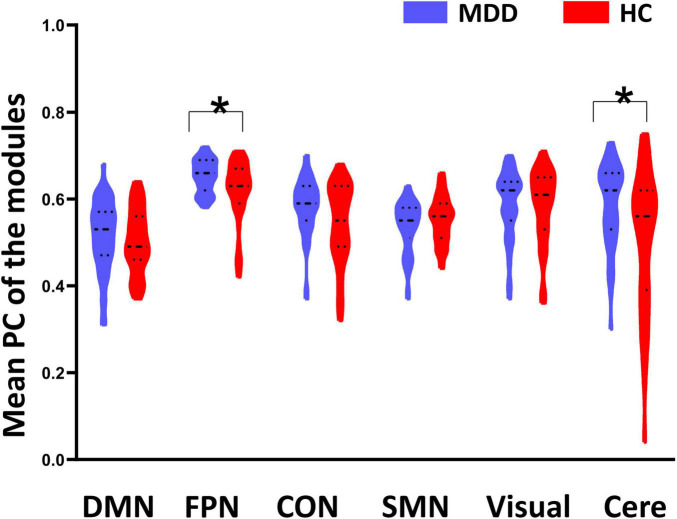
Between-group differences of mean participant coefficient (PC). Major depressive disorder (MDD) patients showed significantly higher PC on the fronto-parietal network (FPN) and cerebellum (Cere) than the healthy controls (HC). *Indicates *p* < 0.05.

**FIGURE 3:**
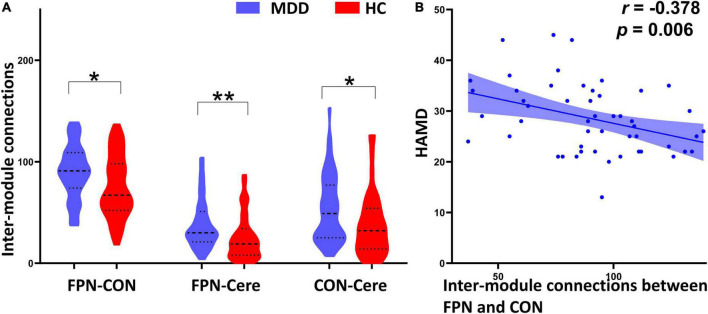
Between-group comparison of inter-module connections and their correlation with HAMD scores. **(A)** Compared with the healthy controls (HC), the major depressive disorder (MDD) patients exhibited significantly increased inter-module connections between the frontal–parietal network (FPN) and cingulo-opercular network (CON), FPN and cerebellum (Cere), CON and Cere. **(B)** The inter-module connections between the FPN and CON are significantly negatively correlated with HAMD scores in the MDD group. *Indicates *p* < 0.05, **indicates *p* < 0.01.

As shown in Figure 1B, the whole brain was parcellated into six functional modules including the DMN, FPN, CON, SMN, Visual, and Cere, which have been broadly applied in prior works. We examined the PC of each module in the MDD and HC groups (Figures 1C,D). MDD patients showed significantly higher mean PC of the FPN (*t* = 2.826, *p*_*corrected*_ = 0.036) and Cere (*t* = 2.796, *p*_*corrected*_ = 0.042) compared with the HC group (Figure 2 and Table 2). In addition to PC, we also determined whether these changers mentioned above were caused by abnormal connections of intra-module and inter-module (Figure 1E). As shown in Figure 3A and Table 2, MDD displayed significantly increased inter-module connections between the FPN and CON (*t* = 2.423, *p _unc_*_*orrected*_ = 0.017), FPN and Cere (*t* = 2.764, *p _unc_*_*orrected*_ = 0.007), CON and Cere (*t* = 2.335, *p _unc_*_*orrected*_ = 0.022) relative to the HC group. No significant between-group difference was observed in the intra-module connections. Correlation analysis revealed that the inter-module connections between the FPN and CON were significantly negatively correlated with HAMD scores (*r* = −0.378, *p _unc_*_*orrected*_ = 0.006) in MDD (Figure 3B and Table 2).

**TABLE 2:** Significant differences in the mean PC of modules, the inter-module connections and the PC of nodes.

	MDD (Mean ± *SD*)	HC (Mean ± *SD*)	*t*-value	*P*-value
**Mean PC of the modules**				
FPN	0.654 ± 0.038	0.619 ± 0.073	2.826	0.036
Cere	0.588 ± 0.094	0.509 ± 0.162	2.796	0.042
**Inter-module connections**				
FPN and CON	89.900 ± 26.837	75.440 ± 31.031	2.423	0.017
FPN and Cere	38.180 ± 23.936	24.950 ± 22.086	2.764	0.007
CON and Cere	53.270 ± 32.959	37.700 ± 31.309	2.335	0.022
**PC of the nodes in FPN**				
R aPFC	0.615 ± 0.110	0.559 ± 0.154	2.039	0.044
L aPFC	0.641 ± 0.106	0.563 ± 0.150	2.868	0.005
L ACC	0.685 ± 0.079	0.619 ± 0.135	2.903	0.005
R IPL	0.636 ± 0.084	0.514 ± 0.230	3.298	0.002
L IPS	0.688 ± 0.079	0.623 ± 0.143	2.648	0.010

MDD, major depressive disorder; HC, healthy controls; SD, standard deviation; PC, participation coefficient; FPN, fronto-parietal network; CON, cingulo-opercular network; Cere, cerebellum; R, right; L, left; aPFC, ventral anterior prefrontal cortex; ACC, anterior cingulate cortex; IPL, inferior parietal lobule; IPS, intraparietal sulcus.

**FIGURE 4:**
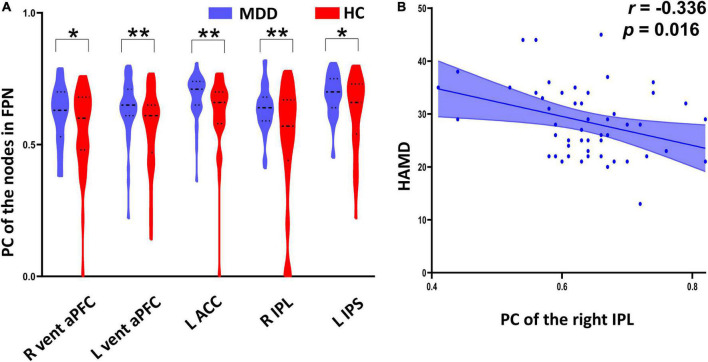
Between-group differences of participation coefficient (PC) of the nodes in frontal–parietal network (FPN) and their correlation with HAMD scores. **(A)** The major depressive disorder (MDD) patients exhibited significantly increased PC in the bilateral ventral anterior prefrontal cortex, left anterior cingulate cortex, right inferior parietal lobule, and left intraparietal sulcus relative to the healthy controls (HC). **(B)** The PC values of the right inferior parietal lobule were significantly negatively correlated with HAMD scores in the MDD group. *Indicates *p* < 0.05, **indicates *p* < 0.01.

As shown in Figure 4A and Table 2, MDD patients exhibited significantly increased PC in the right ventral anterior prefrontal cortex (aPFC, *t* = 2.039, *p _unc_*_*orrected*_ = 0.044), left aPFC (*t* = 2.868, *p _unc_*_*orrected*_ = 0.005), left anterior cingulate cortex (ACC, *t* = 2.903, *p _unc_*_*orrected*_ = 0.005), right inferior parietal lobule (IPL, *t* = 3.298, *p _unc_*_*orrected*_ = 0.002), and left intraparietal sulcus (IPS, *t* = 2.648, *p _unc_*_*orrected*_ = 0.010) compared with the HC group. The PC values of the right IPL were significantly negatively correlated with HAMD scores (*r* = -0.336, *p _unc_*_*orrected*_ = 0.016) in the MDD group (Figure 4B and Table 2).

We selected a range of network density to evaluate the reliability of our findings found above. The threshold range used was 0.10 (10% has been proved to provide high test-retest reliability of graph theory metrics) to 0.20 (20% can potentially reveal the cognitive-relevance of weak brain connections) with a step size of 0.02. Most of the results reported above were retained (Supplementary Tables 1, 2).

## Discussion

In the present study, we delineated the modular segregation patterns of the brain networks by applying graph theory analysis in the MDD and healthy participants, and then examined the relationship between the graph theory properties and depression symptom. We found significantly lower modular segregation in FPN and Cere in the MDD group than in the HC group. MDD patients exhibited increased inter-module connections between the FPN and CON, FPN and Cere, CON and Cere compared with the HC group. PC in the aPFC, ACC, IPL, IPS of MDD patients were increased than that of HC group. Moreover, the inter-module connections between the FPN and CON and the PC values of the IPL were negatively correlated with depression symptom in MDD. These findings extend our understanding of disrupted functional brain networks underlying MDD from a modular perspective.

We found MDD patients had significantly increased PC of the FPN and Cere compared with the HC group, suggesting lower intra-modular connections and higher inter-modular connections in MDD. The FPN regulates the top-down process of emotion and attention (36). Previous studies frequently reported that atypical connectivity of the FPN was linked to MDD (31, 37). Luo et al. have observed lower connectivity within the FPN and higher connectivity between the FPN and other brain regions in MDD group, which indicated alterations of the FPN play a crucial role in the pathophysiology of MDD (31). Furthermore, we also found abnormalities of the Cere in MDD. Many studies have focused increasingly on cerebellar role in delineating the neural loops of MDD. Prior study implies Cere is involved in regulation of emotion process *via* communications with limbic regions (38). Another study demonstrated that the cerebellar connections with DMN might modulate self-reference process (39). Our findings emphasized cerebellar contributions to MDD and further investigations are expected to explore its brain mechanism.

Further analysis suggested that the decreased modular segregations of the FPN and Cere resulted from the increased inter-modular connections between the FPN and CON, FPN and Cere, CON and Cere. The FPN and CON are task-positive networks and modulate processing of emotion and attention (40). In line with our current findings, previous study found participants with MDD had aberrant connectivity between the FPN and CON and correlated with dysregulation of emotion and attention (41). Furthermore, abnormal connectivity between the FPN and CON may contribute to dysfunction of executive control (42, 43). Accordingly, abnormalities between the FPN and CON may underlie depression-related dysregulation of emotion and poor concentration, as well as executive control deficits.

Interestingly, correlation analysis showed that the inter-modular connections between the FPN and CON were negatively correlated with depression symptom, whereas the MDD group displayed higher inter-modular connections between the FPN and CON relative to HC group, exhibiting two opposite tendencies. A large number of neuroimaging studies have demonstrated that MDD not only affects long-distance connections, but also affects local spontaneous neural activity in the brain (44–46). It is worth noting that long-distance connections and local brain neural activities often occur simultaneously and interact (47, 48). In addition, previous studies have documented that depression symptoms are negatively correlated with local neural activity in the brain (49, 50). Therefore, the inter-modular connections between the FPN and CON that decrease with the increase in depressive symptoms may reflect the decrease in local neural activity in the brain, rather than long-distance abnormal changes.

At the nodal level, the between-group comparison showed that the PC in the aPFC, ACC, IPL, and IPS significantly increased in MDD group compared with the HC group. These nodes participate in these functions *via* coupling with other modules. For example, previous structural and functional neuroimaging studies have confirmed that aPFC modulates high-order cognitive function through connections with lateral parietal cortex (51, 52). Many studies have found that subjects with MDD had aberrant activation in the ACC under the emotional tasks (53–55). Rive et al. reported hyperactivation in the ACC during negative tasks and hypoactivation during positive tasks (56). Moreover, Fournier et al. observed that MDD patients exhibited hyperactivation in the ACC when confronted with anger stimuli (57). IPL has been confirmed to be involved in emotional perception. Wang et al. observed that participants with MDD displayed significantly decreased local spontaneous neural activity in bilateral IPL compared to HC group (58). Therefore, current results of increased PC in the ACC and IPL may link to emotional deficits of MDD patients. IPS is necessary for attention control and memory processing. Previous studies on MDD frequently reported IPS alterations. Martin et al. revealed increased activity in the IPS and IPS exerted a negative modulation on visual cortex (59). Fairhall et al. documented dissociation between IPS and hippocampus (60).

There are several limitations in our present study. Firstly, the number of nodes in a module will affect the PC value, so more advanced methods should be designed to calculate PC. Secondly, our results did not find abnormal modular segmentation about DMN, which is often reported in previous network studies on MDD. Further studies are expected to give reasonable explanations to our findings in the current study. Thirdly, only Dosenbach’s template was chosen for network construction, and future research should choose different templates to test the reproducibility of our results.

## Conclusion

Using graph theory analysis, the present study demonstrated decreased modular segregation of the FPN and Cere, which was resulted from the increased inter-modular connections between the FPN and CON, FPN and Cere, CON and Cere. Moreover, we found significantly increased PC in the aPFC, ACC, IPL, and IPS. The above findings explain the emotional dysregulation, cognitive deficits and inattention related to depression from the perspective of modular segregation.

## Data availability statement

The raw data supporting the conclusions of this article will be made available by the authors, without undue reservation.

## Ethics statement

The studies involving human participants were reviewed and approved by the Local Institutional Review Board of Hangzhou Normal University. Written informed consent to participate in this study was provided by the participants’ legal guardian/next of kin.

## Author contributions

ZL, WZ, DW, and S-WX: conceptualization, methodology, formal analysis, writing – original draft preparation, and visualization. YW, CP, YX, and CK: data curation, investigation, and resources. ZT: validation and project administration. DW, S-WX, and WZ: funding acquisition, writing – review and editing, and supervision. All authors contributed to the article and approved the submitted version.

## Conflict of interest

The authors declare that the research was conducted in the absence of any commercial or financial relationships that could be construed as a potential conflict of interest.

## Publisher’s note

All claims expressed in this article are solely those of the authors and do not necessarily represent those of their affiliated organizations, or those of the publisher, the editors and the reviewers. Any product that may be evaluated in this article, or claim that may be made by its manufacturer, is not guaranteed or endorsed by the publisher.

## Footnotes

1
http://www.rfmri.org/
