# Supplementary material for: Decreased modular segregation of the frontal–parietal network in major depressive disorder
Source: Front Psychiatry. 2022 Jul 22;13:929812. doi: 10.3389/fpsyt.2022.929812 (PMC9353222; doi:10.3389/fpsyt.2022.929812)
Supplement: Supplementary file 1 [file Data_Sheet_1.docx]

**Supplemental Material for:**

**Decreased modular segregation of the frontal–parietal network in major depressive disorder**

**Zhihui Lan, Wei Zhang, Donglin Wang, Zhonglin Tan, Yan Wang, Chenyuan Pan, Yang Xiao, Changxiao Kuai, Shao-Wei Xue**

**Supporting Document:**

**Supplementary Table 1 Comparison results under different network densities**

**Supplementary Table 2 The relationship between the inter-module connections of the FPN and CON /the PC values of the IPL and HAMD scores under different network densities**

**Supplementary Table 1** **Comparison results under different network densities**

|  |  | **Network density** | | | | | |
| --- | --- | --- | --- | --- | --- | --- | --- |
|  |  | 0.10 | 0.12 | 0.14 | 0.16 | 0.18 | 0.20 |
| PC | FPN | *t* = 2.763^**^ | *t* = 3.036^***^ | *t* = 3.016^***^ | *t* = 2.957^***^ | *t* = 3.036^***^ | *t* = 3.155^***^ |
|  | Cere | *t* = 2.805^**^ | *t* = 3.022^***^ | *t* = 2.969^***^ | *t* = 2.941^***^ | *t* = 2.944^***^ | *t* = 2.856^**^ |
| Inter-conn | FPN-CON | *t* = 2.498^*^ | *t* = 2.449^*^ | *t* = 2.350^*^ | *t* = 2.346^*^ | *t* = 2.388^*^ | *t* = 2.459^*^ |
|  | FPN-Cere | *t* = 2.411^*^ | *t* = 2.702^**^ | *t* = 2.735^**^ | *t* = 2.735^**^ | *t* = 2.770^**^ | *t* = 2.809^**^ |
|  | CON-Cere | *t* = 2.130^*^ | *t* = 2.139^*^ | *t* = 2.251^*^ | *t* = 2.430^*^ | *t* = 2.483^*^ | *t* = 2.370^*^ |
| PC of nodes | R aPFC | *t* = 1.483 | *t* = 2.730^**^ | *t* = 1.928 | *t* = 2.133^*^ | *t* = 2.144^*^ | *t* = 2.097^*^ |
|  | L aPFC | *t* = 1.958^*^ | *t* = 2.456^*^ | *t* = 2.083^*^ | *t* = 2.919^***^ | *t* = 2.958^***^ | *t* = 3.341^***^ |
|  | L ACC | *t* = 3.658^***^ | *t* = 3.319^**^ | *t* = 3.134^**^ | *t* = 3.055^***^ | *t* = 2.679^**^ | *t* = 2.509^*^ |
|  | R IPL | *t* = 2.645^*^ | *t* = 3.117^**^ | *t* = 3.409^***^ | *t* = 3.280^***^ | *t* = 3.240^***^ | *t* = 3.207^***^ |
|  | L IPS | *t* = 1.445 | *t* = 2.369^*^ | *t* = 2.631^*^ | *t* = 2.992^***^ | *t* = 2.517^*^ | *t* = 2.129^*^ |

**p* _uncorrected_ < 0.05, ***p* _uncorrected_ < 0.01, ****p* _uncorrected_ < 0.005

**Supplementary Table 2** **The relationship between the inter-module connections of the FPN and CON /the PC values of the IPL and HAMD scores under different network densities**

|  |  | **Network density** | | | | | |
| --- | --- | --- | --- | --- | --- | --- | --- |
|  |  | 0.10 | 0.12 | 0.14 | 0.16 | 0.18 | 0.20 |
| Correlations | FPN-CON | *r* = -0.378^**^ | *r* = -0.381^**^ | *r* = -0.398^***^ | *r* = -0.366^**^ | *r* = -0.354^**^ | *r* = -0.388^***^ |
|  | R IPL | *r* = -0.336^*^ | *r* = -0.364^**^ | *r* = -0.364^**^ | *r* = -0.307^*^ | *r* = -0.329^*^ | *r* = -0.275^*^ |

**p* _uncorrected_ < 0.05, ***p* _uncorrected_ < 0.01, ****p* _uncorrected_ < 0.005
